# Muriate of Ammonia in Iritis

**Published:** 1866-02

**Authors:** Jos. S. Hildreth

**Affiliations:** Chicago, late Surgeon in charge of the Desmarres (U.S. Army) Eye and Ear Hospital


ARTICLE VIII.

### MURIATE OF AMMONIA IN IRITIS.

By JOS. S. HILDRETH, M.D., Chicago, late Surgeon in charge of the Desmarres (U.S. Army) Eye and Ear Hospital.

Iritis, like pneumonia, is regarded by some authors as a self-limited affection; which theory is undoubtedly correct, if certain indications are fulfilled by treatment, otherwise, the tendency is to form mechanical and pathological complications that may compromise the eye itself. Though the exciting cause may be rheumatic, syphilitic, or scrofulous, yet something more than treatment of the general disorder will be required. The local danger, first, must be controlled, and, at the same time, as far as practicable, the existing general disturbance. The eye relieved, constitutional treatment next demands attention.

To understand the pathology, and employ the means most favorable to successful treatment of this affection, therefore, becomes of the highest importance. The subject may be considered under three heads:—

1st. Prominent characteristics of iritis, viz.:—

Change of color, swelling, and injection of the iris.

Diminution, or loss of movement of the pupil, which frequently becomes small, irregular, and rough.

Exudations between iris and capsule, into the pupil, and often upon the surface of the iris.

Hypopion, and epauchement of blood into anterior chamber.

Loss, more or less marked, of transparency of cornea, and aqueous humor.

Injection of vessels of sclerotic and conjunctiva around the cornea.

Pain in the eye, in and about the orbit, especially at night.

Fever, headache, insomnia, and other general disturbances may be well marked, or entirely absent.

2d. Prominent effects to be obtained in treatment, viz.:—

Dilate, and so maintain, the pupil until the termination of the disease.

Prevent hypopion, exudations, and epauchements, and promote their absorption when existing.

Facilitate resolution of the congestion and inflammation of the parts.

Relieve the local and other pain and disturbance of the system.

3d. Treatment.

Without dwelling upon the various means that have been, and are, employed in healing this affection, I shall pass at once to those I have found most beneficial.

If the local congestion be great, and the state of the patient will allow it, apply from six to twelve leeches to the temples, or cups.

Instillations of atropia (gr. iv, ℥j,) two or three drops every fourth or sixth hour, until the pupil dilates, if there be no mechanical obstacle; after it is dilated, one drop night and morning, and, finally, one drop daily, to the decline of the disease, will be sufficient. In some cases, more frequent instillations may be found necessary, or the strength of the solution can be increased. But care must be taken to avoid *atropinism* and excitement of the conjunctiva.

Should the state of the gastro-intestinal canal require, give a suitable cathartic. If the stomach be very irritable, it must be allayed by appropriate means.

Morphia, codein, or extract of Indian hemp, the first in $\frac{1}{4}$ grain, the second in $\frac{1}{2}$ grain, and the last in grain doses, to allay pain, if necessary.

Exhibition of muriate of ammonia, as follows:—

If the eye, or parts about it, be painful, especially when local depletion and atropia fail to afford relief, 20 grains of muriate of ammonia may be given every half-hour, till one or two drachms have been taken. This modifies, and frequently controls, these symptoms. Congestive cephalalgia requires similar, and nausea small, doses.

If the pain is not relieved, or only partially so, narcotics should be given *pro re nata*, and the use of muriate of ammonia, in two grain doses, continued every two hours, except at night, or oftener, according to the gravity of the symptoms, until the acute stage has passed, after which two or three times daily will be sufficient.

Cases not accompanied by pain should be treated, from the commencement, by small doses. If the throat be dry, add one grain of chlorate of potassa.

Excessive transpiration must be controlled by appropriate means.

For nocturnal pain, administer large doses every half hour, as described above, accompanied or followed by narcotics, if necessary.

When leeching is inadmissible, and, in absence of mechanical causes, the pupil refuses to dilate, though there be no pain, commence with large doses also.

The following mixture is usually acceptable:—

℞. Ammoniæ Muriat., ---------------------- ℥j.

Syrupi Auranti, ---------------------- ℥ss.

Aquæ, ---------------------------------- ℥iijss.

M.S.A. Sig. one teaspoonful.

Larger quantities had better be administered in enough pure water to avoid irritation of the stomach.

In chronic cases, two grains, three times daily, will be sufficient. With syphilitic complications, the bichloride of mercury; with rheumatic and scrofulous, colchicum, cod liver oil, and other suitable remedies may be given at the same time.

Under its influence, engorgement of the iris is diminished; absorption of hypopion, sanguineous epauchement, and exudation promoted; and neuralgic symptoms modified. Fever, heavy and painful sensations of the head, scantiness of urine, and constipation are controlled. Nausea is usualy relieved, and the effects of narcotics would, in some cases, seem to be promoted.

The action of this salt, in the treatment of iritis, may be summed up as follows:—

*a.* Large doses modify neuralgic pain, congestive cephalalgia, and frequently act as a laxative.

*b.* Small doses exert an influence on the capillary, glandular, and lymphatic systems which facilitates dilatation of the pupil, resolution of the inflammation, absorption of exudations, hypopion, etc.

*c.* It allays nausea, unless the dose be too large, or the stomach very irritable.

*d.* Fever, coated tongue, and other constitutional disturbances are relieved.

*e.* It appears to facilitate the action of narcotics, and produces no irritation of the conjunctiva.

The value of mercury, quinia, iodide of potassium, oil of turpentine, and some other remedies, in the treatment of this affection, is to well known to require comment. Yet, from no single remedy, except belladonna, have I been able to obtain so satisfactory results, as from the one specially dwelt upon in this article.

Its admissibility in many conditions of the system, wide range of action, facility and safety of administration render it peculiarly applicable to this class of cases.

The bromide of ammonia has, in some respects, properties quite similar.

From 30 to 60 grains, well diluted, exert a decided hypnotic action, and small quantities produce some of the effects of the muriate. For certain patients, I think the former preferable in large doses to the latter; but as a resolvent, present experience favors the *chlorine* salt.

Thorough and carefully conducted clinical observations, of over three years, have demonstrated the truth of the above. During this period, every known form of the disease has fallen under my care, whilst military discipline has afforded an opportunity to follow each case from the commencement of treatment to its close.

*Feb.* 5, 1866.

